# CO_2_ emission accounts of Russia’s constituent entities 2005–2019

**DOI:** 10.1038/s41597-021-00966-z

**Published:** 2021-07-13

**Authors:** Huijuan Xiao, Weichen Zhao, Yuli Shan, Dabo Guan

**Affiliations:** 1 Department of Industrial and Systems Engineering, The Hong Kong Polytechnic University, Hong Kong, China; 2 Department of Earth System Science, Tsinghua University, Beijing, 100084 China; 3 Integrated Research on Energy, Environment and Society (IREES), Energy and Sustainability Research Institute Groningen, University of Groningen, Groningen, 9747 AG the Netherlands; 4 The Bartlett School of Sustainable Construction, University College London, London, WC1E 7HB UK

**Keywords:** Climate change, Environmental impact

## Abstract

Constituent entities which make up Russia have wide-ranging powers and are considered as important policymakers and implementers of climate change mitigation. Formulation of CO_2_ emission inventories for Russia’s constituent entities is the priority step in achieving emission reduction. Russia is the world’s largest exporter of oil and gas combined and the fourth biggest CO_2_ emitter, so it’s efforts in mitigating CO_2_ emissions are globally significant in curbing climate change. However, the existing emission inventories only present national CO_2_ emissions; the subnational emission details are missing. In addition, the emission factors are not country-specific and energy activity data by fossil energy types and sectors are not sufficiently detailed. In this study, the CO_2_ emission inventories of Russia and its 82 constituent entities from 2005 to 2019 are constructed. The emission inventories include energy-related emissions with 89 socio-economic sectors and 17 energy types and process-related emissions. The uniformly formatted emission inventories can be a reference for in-depth analysis of emission characteristics and emission-related studies of Russia.

## Abstract (summary)

**Table:** 

Measurement(s)	carbon dioxide emission
Technology Type(s)	digital curation
Factor Type(s)	sub-national constituent entity • year
Sample Characteristic - Environment	climate system
Sample Characteristic - Location	Russia

Machine-accessible metadata file describing the reported data: 10.6084/m9.figshare.14798694

## Background & Summary

Global climate change has increased risks and impacted on many aspects of nature and human life, such as rising sea levels for low-lying areas, loss of marine species, eroding food security and slowing down economic growth^1,2^. Skyrocketing levels of greenhouse gas in recent decades is the main cause of global climate change^3^. Russia is the fourth largest emission contributor in the world, generating up to 1536.9 million tonnes in 2017^4^. Also, because of Russia’ s abundant natural resources, it is not only the third largest oil producer but the second largest gas producer, representing around 12.1% and 17.3% of the world output in 2018, respectively^5^. Russian oil exports and gas exports accounted for 12.8% and 26.3% of the global total in 2018, respectively^5^. Considering Russia’s vast territorial size, large population, energy-intensive economic activities and the important role of fossil fuel production, it could play an important role in mitigating CO_2_ emissions and putting the brakes on global climate change. In 2019, Russia formally joined the Paris agreement, which aims to enhance international cooperation to mitigate global climate change^6^.

Russia is composed of oblasts, republics, krais, autonomous okrugs, federal cities and autonomous oblasts, which are equal constituent entities of Russia^7^. As a transcontinental country, Russia stretches across a large expanse of Northern Asia and Eastern Europe. Because constituent entities are different in natural resource endowments, industry structure and socioeconomic development stages^7–10^, each constituent entity should have targeted emission mitigation strategies which are designed according to constituent entities’ unique emission characteristics. Constituent entities have wide-ranging powers and are considered as important policymakers and implementers of climate change mitigation. An accurate formulation of a CO_2_ emission inventory for the constituent entities is the priority step in achieving emission reductions in of Russia. Many international institutes also estimated national CO_2_ emissions, including the International Energy Agency (IEA)^4^, Carbon Dioxide Information Analysis Center (CDIAC)^11^, Energy Information Administration (EIA), Emission Database for Global Atmospheric Research (EDGAR)^12^ and British Petroleum (BP)^5^. However, the existing emission inventories only measure CO_2_ emissions at national level, with subnational emission details missing. The absence of emission data at the subnational level creates a barrier to an in-depth analysis of emission characteristics and targeted mitigation strategies.

As for the emission factors used to calculate CO_2_ emissions, Russia’s emission accounting is generally based on the default emission factors recommended by the Intergovernmental Panel on Climate Change (IPCC)^13^, which are not country-specific and not representative enough. Also, CO_2_ emissions by fossil energy types and sectors are not sufficiently detailed. Some of them only provide Russia’s total emissions, or at best for some key sectors and fossil fuel types. For example, BP only provides the total amount of CO_2_ emissions of Russia and the IEA provides emissions only from four energy types (coal, oil, natural gas and other) and nine sectors^4,5^.

Considering the large emission data gap at subnational level and sketchy national data, our dataset includes the CO_2_ emission inventories of 82 constituent entities and Russia between 2005 and 2019. The emission database is constructed according to detailed socioeconomic sectors and energy types in a uniform format, which presents emissions from 17 energy types and 89 socio-economic sectors. Also, the emission construction method of the 82 constituent entities is consistent with the method of national estimation, which enables multi-scale emission studies and increases comparability. The emission inventories will be updated and published yearly. Our emission inventory is constructed based on country-specific emission factors provided by the Ministry of Natural Resources and Environment (MNRE) of Russia^14^. These emission datasets can provide robust data support for follow-up studies of Russia’s emission-related issues and formulation of decarbonization strategies. The emissions dataset can be accessed freely from the China Emission Accounts and Datasets (CEADs, www.ceads.net).

## Methods

**Fig. 1:**
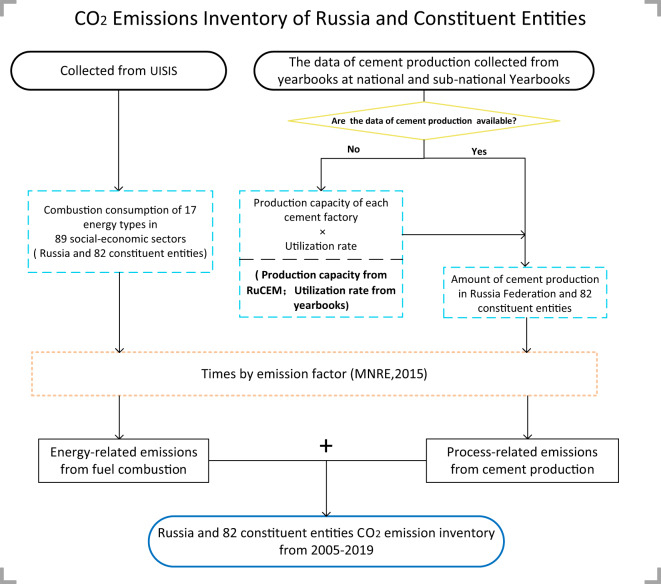
The framework of CO_2_ emissions inventory construction.

In general, CO_2_ emissions accounting includes three scopes^15^. Scope 1 indicates direct CO_2_ emissions generated within a territory, which is also known as territorial-based emissions. Scope 1 accounts for all CO_2_ emissions produced within a region boundary, such as emissions from local energy production enterprises^16,17^. Scope 2 indicates indirect CO_2_ emissions embodied in electricity, steam and heat imported from another territory^15,18^. Scope 3 indicates indirect CO_2_ emission embodied in products and services which are imported from another territory^15,18^. The compilation of CO_2_ emissions inventory was constructed according to the IPCC administrative territorial-based accounting scope, that is Scope 1^13^. The impact of international aviation and shipping is not included in our estimation^19^. CO_2_ emission inventories consist of two components, as shown in Fig. 1: energy- and process-related (cement) CO_2_ emissions^20–22^. The energy-related emissions suggest the CO_2_ emissions generated when burning the fossil fuel^23–25^. Process-related emissions indicate CO_2_ emissions produced during the chemical reactions of the industrial process, with the CO_2_ emissions converted from industrial raw materials, rather than fossil fuels^23,26,27^. For example, calcium carbonate can be converted to get CO_2_ emissions and calcium oxide when producing cement^28–30^.

### Energy-related CO_2_ emissions

Energy-related CO_2_ emissions are constructed as follows^13,31^.

1 $${\rm{C}}{E}_{ij}=\mathop{\sum }\limits_{i=1}^{17}\mathop{\sum }\limits_{j=1}^{89}A{D}_{ij}\times NC{V}_{i}\times C{C}_{i}\times {O}_{ij}$$

In Eq. (1), *i* and *j* indicate energy types and socio-economic sectors, respectively. *CE*_*ij*_ indicates CO_2_ emissions from fossil fuel *i* combusted in sector *j*. *NCV*_*i*_ is net caloric value, indicating the heat produced per physical unit of fossil fuel during the combustion process. *CC*_*i*_ means carbon content per calorie of fossil fuel. *O*_*ij*_ indicates carbon oxidation ratio, which is the percentage of carbon converted to CO_2_ emissions in fossil fuel. *AD*_*ij*_ indicates activity data. As for energy-related emission accounting, *AD*_*ij*_ refers to the amount of fossil fuel used for combustion.

Most of the studies and international institutes adopted the default emission factors provide by the IPCC. This study adopts the emission factors from the MNRE of Russia^14^. Compared with emission factors from the IPCC, country-specific emission factors measured by the MNRE are more representative of the fossil fuel situation in Russia. For example, the MNRE released the emission factors of 29 types of coal based on their mining areas, as shown in Table 1. Because of Russia’s large territory, the quality of coal differs significantly among regions, such as Kuznetskiy basin, Donetskiy basin and Kansk-Achinskiy basin, and their emission factors range from 0.73 tonne CO_2_/tonne to 2.72 tonne CO_2_/tonne (shown in Table 1). However, the default value of coal issued by the IPCC is around 2.61 tonne CO_2_/tonne. The differences between emission factors provided by the IPCC and the MNRE of Russia are illustrated in Table 1. Among all the fossil fuels, the emission factor of blast furnace gas shows the largest gap evaluated by the MNRE (3.28 tonne CO_2_/tonne) and the IPCC (0.76 tonne CO_2_/tonne). We also compared the level of CO_2_ emissions evaluated based on MNRE, IPCC and two other sources and explained the difference in section 4.2.

**Table 1:** Comparisons of CO_2_ emission factors of fossil fuels (tonne CO_2_/tonne, 1000 m^3^).

No.	Fuel types	MNRE-this study	IPCC	No.	Fuel types	MNRE-this study	IPCC
1	Gasoline	3.00	3.05	17.7	Intaugol coal	1.77	—
2	Fuel oil	3.08	3.09	17.8	Kamchatka Krai coal	0.88	—
3	Diesel	3.12	3.15	17.9	Kansk-Achinskiy coal	1.48	—
4	Bunker fuel	3.10^1^	3.12^1^	17.1	Karaganda coal	2.01	—
5	Marine fuel	3.21	3.33^1^	17.11	Kuznetskiy coal	2.33	—
6	Liquefied propane and butane	2.88	2.86	17.12	Magadan coal	1.91	—
7	Other petroleum products	3.04	2.90	17.13	Neryungrinsky coal	2.72	—
8	Artificial coke gas	0.74	0.83	17.14	Norilsk coal	2.10	—
9	Blast furnace gas	3.28	0.76	17.15	Moscow coal	0.93	—
10	Combustible natural gas	1.83	1.91	17.16	Primorye coal	1.38	—
11	Associated petroleum gas	2.02	2.95	17.17	Coal from other deposits	2.12	—
12	Fuelwood	0.69	1.22	17.18	Raichikhinsky coal	2.12	—
13	Household boiler fuel	3.26	3.11	17.19	Sakhalin coal	2.12	—
14	Peat	1.04	1.03	17.20	Sverdlovsk coal	0.91	—
15	Peat briquettes and semi-briquettes	1.83	1.03	17.21	Ulug-Khem coal (The Tyva Republic)	2.51	—
16	Other solid fuel	1.39^2^	1.16	17.22	Tunguska coal	2.08	—
17	Coal:	—	2.61	17.23	Urgal coal	2.12	—
17.1	Azeyskoye coal	1.33	—	17.24	Minusinsk coal (Republic of Khakassia)	2.01	—
17.2	Bashkir coal	0.73	—	17.25	Chelyabinsk coal	1.54	—
17.3	Vorkuta coal	2.23	—	17.26	Yakutskiy coal	2.07	—
17.4	Gusinoozyorsk coal	1.40	—	17.27	Cheremkhovsky coal	2.07	—
17.5	Donetsk coal	2.32	—	17.28	Chita coal	1.40	—
17.6	Imported coal	2.12	—	17.29	Ekibastuz coal	1.74	—

^1^Emission factors of bunker fuel (MNRE), bunker fuel (IPCC), and marine fuel (IPCC) are based on the weighted average of their main components (fuel oil and diesel) since MNRE and IPCC do not release the related emission factors.

^2^Other solid fuel includes industrial waste, residential waste, and other types of natural fuel (e.g., straw, brushwood, and waste from logging and woodworking). Thus, its emission factor is based on the average emission factor of its components.

The study collects the energy activity data from the Unified Interdepartmental Statistical Information System of Russia (UISIS)^32^. UISIS is the state integrated statistical resource and the largest provider of statistical data in Russia at national and subnational levels. The raw energy data are sourced from the 4-TER form (information on the use of fuel and energy sources) filled out by legal entities of energy consumers and suppliers in Russia (except small enterprises). The completed form is then submitted to the Federal State Statistics Service (Rosstat) of the territorial body where the separate subdivision is located or where the legal entity is located if it does not have a separate subdivision. If a legal entity does not carry out the activities in its location, the form should be submitted at the place where the activities are carried out. Energy activity data includes the energy used for combustion in the final consumption and the energy used for process and transformation (e.g., electricity and heat generation) within the nation/constituent entity boundaries. Emissions generated from imported electricity and heat are not included in this study since we focus on emissions produced within the nation/constituent entity boundary (Scope 1). The Energy activity data provided by UISIS includes total energy consumption, energy used for feedstocks, and energy used for non-fuel needs. A relatively small proportion of energy used for feedstocks and non-fuel needs has been excluded in the calculation of energy-related emissions. Examples about the energy used for feedstocks can be the production of chemical, petrochemical or other non-fuel products. As for the non-fuel needs, they can be the chemical reagents for drilling oil wells, gas injection to maintain reservoir pressure, lubricant, and insulating material. Based on the categorization method of the UISIS^32^, there are 45 types of fossil fuels, which include 29 different types of coal based on their mining areas, as shown in Table 1. In the emission inventory, we merged the CO_2_ emissions from these 29 types of coal into CO_2_ emissions from one energy type, that is coal, due to their similar energy quality and for better demonstration. In other words, this study shows CO_2_ emissions from 17 energy types. Since the unit of fuelwood released by UISIS is in cubic meters^32^, the emission factor of fuelwood provided by the MNRE cannot be directly used to measure CO_2_ emissions. Therefore, we first converted the unit of fuelwood to tonnes by using the density unit provided by the Self-regulatory Organization (SRO) of Russia^33^, at 0.6 tonne/m^3^.

The sector’s classification is according to the document of the Russian Classification of Economic Activities code ОK 029–2014 (OKVED 2 NACE Rev. 2) provided by the Federal Agency for Technical Regulation and Metrology^34^. This is a hierarchical classification method which includes four levels, that is: sections (an alphabetical code), divisions (two-digit numerical code), groups (three-digit numerical code) and classes (four-digit numerical code), as shown in Online-only Table 1. To save space, we do not always show the lower hierarchical levels since not all the sectors generate CO_2_ emissions. In other words, all sections are contained in the emission inventory, while the division, group, and class levels will be included only when this sector generates CO_2_ emissions. Since this study accounts for CO_2_ emissions produced within a region boundary, we excluded a section which does not consume energy activity data within the boundary, that is ‘section U: activities of extraterritorial organization and bodies’.

There are some subsectors, for which UISIS does not provide energy activity data, and this leads to a gap between the main sectors and the summation of its lower level sectors. Considering that this gap does not belong to a specific subsector, we allocated this gap to a newly constructed sector, which is the combination of several subsectors. For example, energy activity data is only available in ‘Q section: Human health and social work activities’ and’No. 86: health service activities’, while the data of’No. 87: Residential care activities’ and’No. 88: social work activities without accommodation’ are not available (Section Q= No. 86+No. 87+No. 88) (shown in Online-only Table 1). Therefore, there will be an emission gap between Q section and No.86 sector, so we combined No.87 and No.88 into one sector, named as ‘social service activities’ and the CO_2_ emissions gap is then allocated to this newly constructed sector (shown in Online-only Table 1). In general, there are 11 newly constructed sectors: ‘Crop production, hunting and related services’, ‘Raising of other animals’, ‘Transmission, distribution and trade of electricity’, ‘Gas distribution and trade’, ‘Transmission, distribution and trade of steam and hot water; Maintenance of thermal network and boiler room’, ‘Construction of other civil projects’, ‘Demolition and site preparation’, ‘Other construction works’, ‘Non-specialized wholesale trade’, ‘Wholesale trade of other specialized products’ and ‘Social service activities’ (shown in Online-only Table 1). Based on the above processes, there are 89 sectors contained in the construction of CO_2_ emissions in this study after excluding the double counting sectors, as shown in Online-only Table 1. For completeness, apart from these 89 sectors, we also demonstrate the CO_2_ emissions of sectors of higher-level classification in the emission inventory. There may still be a small gap between aggregated emissions of subsectors and emissions of their main sector due to measurement errors. To eliminate this gap, we further allocated the small gap to subsectors based on their share of CO_2_ emissions.

### Process-related (cement) CO_2_ emissions

The process-related CO_2_ emissions are calculated in Eq. (2).

2 $$C{E}_{t}=\left\{\begin{array}{ll}Direct\;approach: & EF\times A{D}_{Cement-d}\\ Indirect\;approach: & EF\times A{D}_{Cement-ind}=EF\times PC\times UR\end{array}\right.$$

In Eq. (2), *EF* and *AD* mean and emission factor for cement production released by the MNRE^14^ and activity data (cement production level), respectively. Based on the availability of production data, we adopted two approaches to collect the amount of cement production (*AD*_*cement*_) of 82 constituent entities, that is direct activity data (*AD*_*Cement–d*_) and indirect activity data (*AD*_*Cement–ind*_). *AD*_*cement–d*_ is collected from 82 constituent entities’ yearbooks, however, only five constituent entities released their cement production data, which are Sverdlovsk Region, Chelyabinsk Region, Bryansk Region, Karachayevo-Chircassian Republic, and Krasnodar Territory. For the other constituent entities, the activity data is obtained indirectly (*AD*_*Cement–ind*_.) by multiplying the production capacity data (PC) by utilization rate (UR) of each cement plant. As shown in Fig. 1, we use the point source database of the Russian cement plants from RuCEM^35^, which includes the production capacity of all cement plants in Russia. And then, according to the constituent entities where each cement plant is located, we collected the (UR) of production capacity in these constituent entities, which are available from yearbooks. Therefore, the cement production data of these constituent entities can be obtained by multiplying PC of the cement plant located in each constituent entity by UR in the corresponding year. The CO_2_ emissions from cement production belong to ‘Manufacture of other non-metallic mineral products’ sector, as shown in Online-only Table 1.

Since 2020, yearbooks have not been published officially, only Russia’s national cement production data can be collected in 2019 from CMPRO^36^. We estimate the CO_2_ emissions of the constituent entities in 2019 by downscaling from the national level. The downscale factor is based on the share of the CO_2_ emissions from the cement production of constituent entities in Russia in 2018. We will update the process-related emissions of 82 constituent entities in 2019 once the related data are available.

## Data Records

A total of 2466 data records, including energy-related and process-related emission inventories, are contained in the datasets. The present dataset is made public under Figshare (10.6084/m9.figshare.13084007.v4)^37^. Of these,

- 972 are energy-related emission inventories by energy types for Russia and 82 constituent entitie from 2005 to 2016 [File ‘2005–2016 Energy-related emissions of Russia and 82 constituent entities’]
- 249 are energy-related emission inventories by energy types and by sectors for Russia and 82 constituent entities from 2017 to 2019 [Files ‘2017 Energy-related emissions of Russia and 82 constituent entities’, ‘2018 Energy-related emissions of Russia and 82 constituent entities’, ‘2019 Energy-related emissions of Russia and 82 constituent entities’]
- 1245 are process-related inventories for Russia and 82 constituent entities from 2005 to 2019 [File ‘2005–2019 Process-related emissions (cement) of Russia and 82 constituent entites]

Khanty-Mansi Autonomous Area–Yugra, Yamal-Nenets Autonomous Area, and Tyumen Region less autonomous areas are studied as one (Tyumen Region). Similarly, Nenets Autonomous Okrug and Arkhangelsk Region less autonomous area are also studied as one (Arkhangelsk Region). To sum up, 82 constituent entities are included in this study. Because of Ukraine’s political crisis^38^ and the Chechen-Russian conflict^39^, the inventory data of the Republic of Crimea and Sevastopol city are only available from 2014–2019, and the data of the Chechen Republic only available from 2009–2019. Therefore, a total of 972 energy-related emission inventories from 2005 to 2016 are recorded. As UISIS released the energy activity data from 2005 to 2016 only by energy types and without detailed sectors, the emission inventory from 2005 to 2016 is demonstrated without sectors^40^. From 2016 to 2019, UISIS released the energy activity data in more detail, indicating the dataset is not only by energy types but by sectors^32^. Therefore, the emission inventory from 2016 to 2019 is shown by both energy types and sectors and a total of 249 energy-related emission inventories from 2017 to 2019 are recorded.

**Fig. 2:**
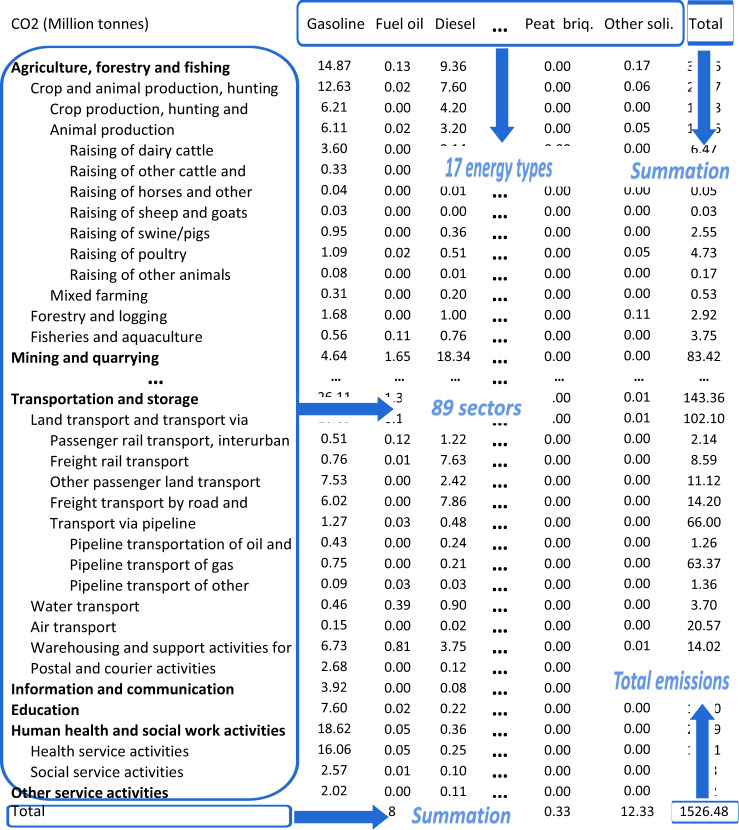
Layout of the CO_2_ emission inventory.

**Fig. 3:**
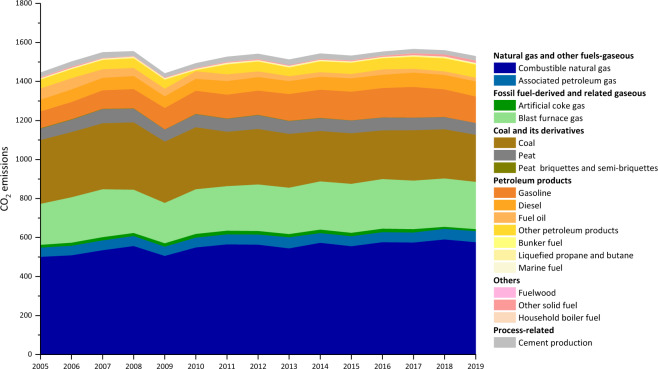
Russia’s CO_2_ emissions 2005–2019, in million tonnes.

The CO_2_ emissions inventories from 2017 to 2019 are matrices with 18 columns and 120 rows, as shown in Figshare^37^. The 18 columns are 17 fossil fuel-related emissions and total emissions (shown in Fig. 2). The 120 rows include 89 sectors and the remaining 31 higher level sectors (shown in Fig. 2). For example, ‘Section Q: Health and social service activities’ is a higher level sector, which includes two subsectors (‘health service activities’ and ‘social service activities’) and we show the data of both the main sector and its subsectors (shown in Fig. 2). Each element of the matrices indicates the CO_2_ emissions from the combustion of a certain energy type in the corresponding sector (shown in Fig. 2). The units of energy-related emissions and process-related emissions provided are million tonnes. As shown in Fig. 3, the stacked area chart represents CO_2_ emissions from 17 fossil fuels combustion and cement production. The chart shows that Russia’s CO_2_ emissions increased in fluctuations from 2005 to 2019, and reached 1549.52 million tonnes in 2019 (shown in Fig. 3). Natural gas is the primary source of CO_2_ emissions from 2005–2019, accounting for about 37.11% of the total (shown in Fig. 3). The proportion of CO_2_ emitted from coal combustion is gradually decreasing, from 22.66% in 2005 to 15.57% in 2019, while the share of CO_2_ emissions produced by the combustion of petroleum products has increased from 17.45% in 2005 to 21.12% in 2019 (shown in Fig. 3). After 2014 the proportion of CO_2_ emissions from petroleum product combustion exceeds that of coal as the second source of CO_2_ emissions (shown in Fig. 3). Overall, Russia’s energy structure is relatively stable from 2005 to 2019 (shown in Fig. 3).

**Fig. 4:**
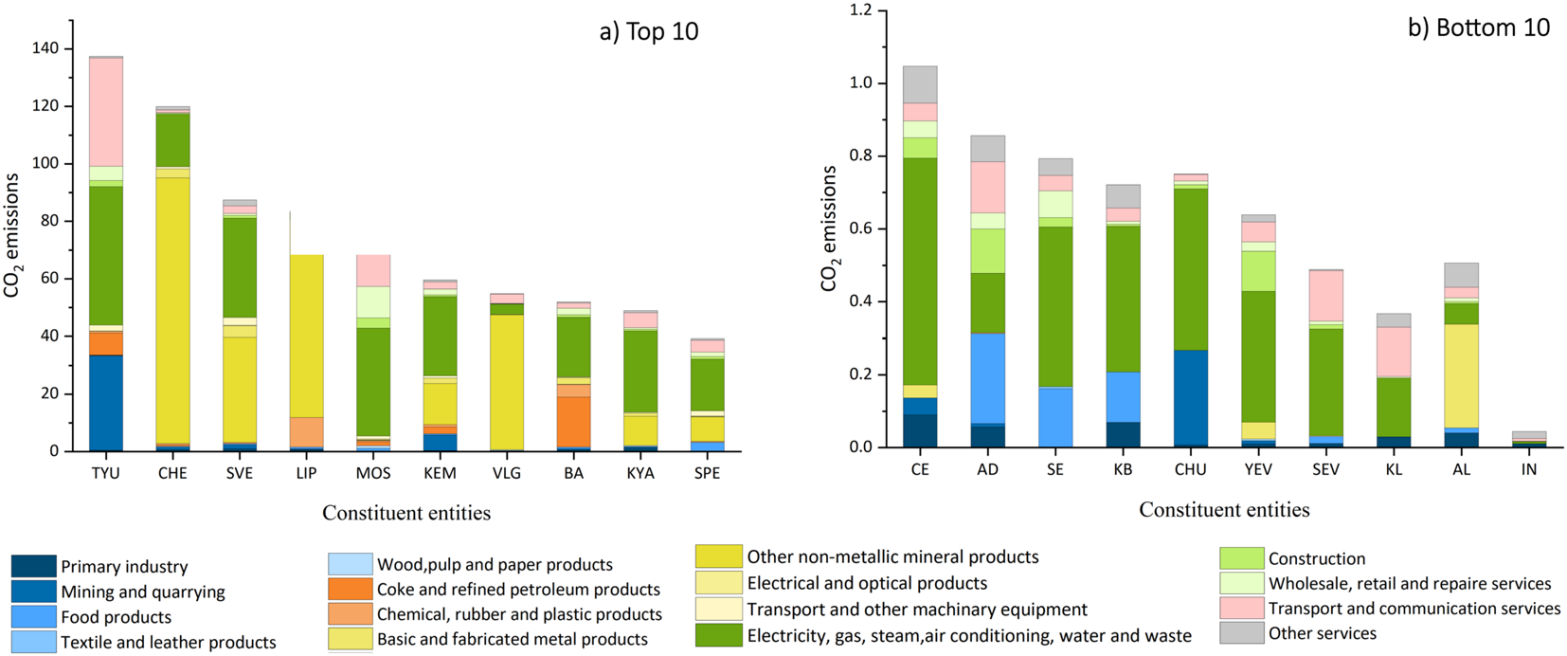
Top/bottom 10 constituent entities in CO_2_ emissions in 2019, in million tonnes. To save space, we use the abbreviation name of the 82 constituent entities based on the standard of International Organization for Standardization (ISO 3166–2) (shown in Online-only Table 2).

Figure 4 presents the CO_2_ emissions of 82 constituent entities by sectors in 2019. The 89 sectors are categorized into 16 main sectors for better demonstration and the categorization details can refer to Online-only Table 1. There was vast regional heterogeneity in CO_2_ emissions among the 82 constituents. From Fig. 4, we find that the Tyumen region is the top emitter among the 82 constituent entities in 2019, contributing around 137.41 million tonnes of CO_2_ emissions. This is mainly because the Tyumen region accounts for more than half of Russia’s production of oil, natural and associated gas^41^. The Chelyabinsk region is the second largest emitter in 2019, generating about 119.96 million tonnes of CO_2_ emissions, primarily because the Chelyabinsk region is one of the oldest mining bases with abundant mineral resources (shown in Fig. 4). Moscow city, the capital of Russia, also produced a relatively large amount of CO_2_ emissions, at around 79.05 million tonnes in 2019 (shown in Fig. 4).

**Fig. 5:**
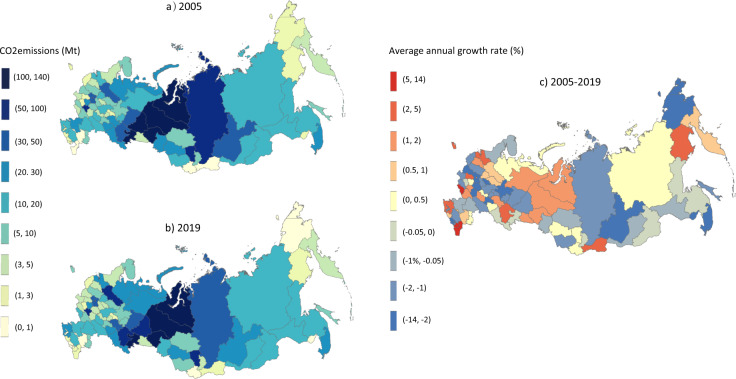
CO_2_ emissions of 82 constituent entities from 2005 to 2019.

The dynamic changes of CO_2_ emissions of 82 constituent entities from 2005 to 2019 can be found in Online-only Table 3 and Fig. 5. Based on the colors shown in Fig. 5, the Tyumen region and the Chelyabinsk region were the top two largest emitters in 2005 and 2019. 37 out of 82 constituent entities experienced an increase during 2005–2019 (shown in Online-only Table 3). The Tyumen region saw the maximum rise in emissions, increasing by 24.26 million tonnes, followed by the Lipetsk region (19.53 million tonnes) and the St. Peterburg city (19.02 million tonnes), the Leningrad_region (13.64 million tonnes), and the Moscow city (13.30 million tonnes) (shown in Online-only Table 3). In contrast, the Sverdlovsk region, the Krasnoyarsk territory, and the Moscow region witnessed the most significant decrease during the study period, dropping by 15.50 million tonnes, 13.61 million tonnes, and 11.43 million tonnes, respectively (shown in Online-only Table 3). For the average growth rate, the CO_2_ emissions of Chukotka autonomous witnessed the fastest decrease between 2005 and 2019, at 8.01% annually (shown in Fig. 5).

## Technical Validation

### Comparisons with existing emission datasets

**Fig. 6:**
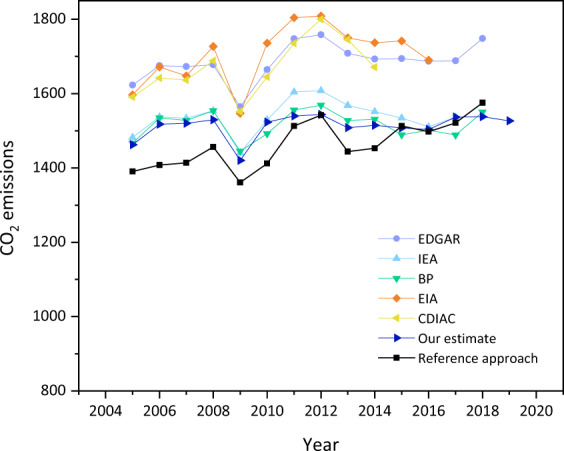
Comparison of Russia’s energy-related CO_2_ emissions with five international institutions, in million tonnes, 2005–2019.

Emission inventories are indispensable in making many environmental decisions and setting scientific mitigation targets. Policy design and emission-related studies require reliable and accurate emission inventories. Since our estimate is based on the 4-TER form covering only large and medium companies, it is important to understand the robustness and accuracy of our emission inventories. Figure 6 shows the comparisons of energy-related CO_2_ emissions of our estimate with the emissions estimated based on the reference approach and five international institutions (EDGAR, IEA, BP, EIA, and CDIAC). Our study is estimated using the sectoral approach, while the reference approach can also be used to calculate the energy-related emissions^24^. The sectoral emissions are calculated from the energy consumption side, while the reference emissions are evaluated based on production side using the energy balance tables (energy consumption = production + import - export - international shipping and aviation - non-energy use, reductants, and feedstocks ± stock change)^24^. Theoretically, the energy data from consumption side and production side should be equal. However, there can be some differences due to many reasons, such as different scopes of statistics and statistical errors. The reference approach is considered to be more accurate for two reasons^42^. First, the reference approach is evaluated according to the fuel production and trade statistics, which are more reliable. Second, the reference approach can avoid accounting errors during the energy processing and conversion process. Therefore, we further compare our estimates with the emission inventories using the reference approach, which are derived from Russia’s national inventory reports (shown in Fig. 6). Results show that the difference between our estimates and the reference approach is relatively small over the study period, at 2.24% on average. This verifies that although our estimate does not cover the small companies, the potential underestimation issue is not significant.

Some differences can also be found when comparing with the emissions presented by five international institutions. The time-series trend of our estimate is consistent overall with other international institutions. For example, there was a sudden decrease in CO_2_ emissions in 2009, and then a rebound can be seen after that (shown in Fig. 6). It can be interpreted by the negative impact caused by the 2008 financial crisis. Our estimate is closer to BP and IEA (shown in Fig. 6). Compared with BP, our estimate shows gaps ranging between 0.82% and 4.01% (shown in Fig. 6). Compared with the IEA, our estimate shows differences ranging between 0.48% and 7.01% (shown in Fig. 6). Since existing emission inventories of Russia do not provide detailed emissions by energy types and socio-economic sectors, a further comparison of the emissions by energy types and by socio-economic sectors cannot be made. In other words, our emission dataset provides the most up to date and comprehensive emission inventories of Russia and its 82 constituent entities and is an important supplement and improvement to the current emission inventories.

### Comparisons with different emission factors

**Fig. 7:**
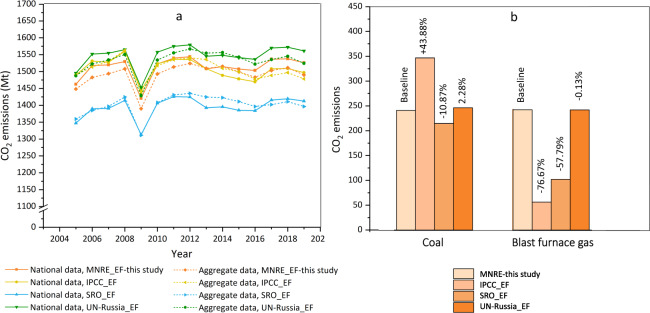
Comparisons of CO_2_ emissions with different emission factor, in million tonnes. The number on the top of the bar chart indicates the percentage change rate compared with baseline in 2019.

We first compare the national CO_2_ emissions (shown in Fig. 7, National data, MNRE_EF) with the aggregation of the 82 constituent entities (shown in Fig. 7, Aggregate data, MNRE_EF). It can be seen that the gap between these two emissions is relatively small, ranging between −1.18 million tonnes and 36.47 million tonnes, representing 0.00% and 0.02% of national CO_2_ emissions. This small gap can be regarded as mutual verification of the quality of energy activity data of both Russia and its constituent entities, which shows the robustness of our estimate. As mentioned above in the Method section, we adopt the country-specific emission factors from the MNRE of Russia^14^. However, the estimation of emission factors provided by different institutions varies, which may lead to different results.

To quantitatively characterize the range of emission factors, this study summarized the emission factors from four sources: MNRE, IPCC, Energy auditor self-regulatory organization (SRO) and United Nations-Russia, as can be found in Online-only Table 4. It shows that among the emission factors of most fossil fuels from four sources, the IPCC has the highest value regarding diesel, artifical coke gas, combustible natural gas, associated petroleum gas, fuelwood, and coal. In terms of the components of emission factors, the net calorific value (NCV) of many emission factors from the IPCC, are higher than the other three sources, especially coal, while the oxygenation efficiency and carbon content are relatively similar. Specifically, the NCV of coal released by the IPCC is 8.83, 8.15 and 10.58 higher than the MNRE, UN-Russia and SRO respectively and the CO_2_ emissions of coal combustion calculated using the IPCC emission factor are 105.83 million tonnes (43.88%), 132.04 million tonnes (61.42%), 100.32 million tonnes (40.67%) higher than the MNRE, the SRO and UN-Russia, respectively (shown in Fig. 7). Additionally, the main types of coal consumed in the Russian Federation come from the Kuznetskiy Basin, the Kansk-Achonskiy Basin and East Siberia^32^, and the NCV of coals in these three places are lower than the IPCC released (shown in Online-only Table 4). For example, the NCV of Kansk-Achonskiy coal is only 15.10 TJ/thousand tonnes, half of that by the IPCC, so the emission factor of coal released by the IPCC is not representative enough. Although, the emission factors of many fossil fuels from the IPCC have the largest value, the CO_2_ emissions calculated adopted the emission factors of the IPCC, which is lower than the UN-Russia and the MNRE, and higher than the SRO (shown in Fig. 7). This is mainly because the NCV of blast furnace gas is only a quarter of the other two sources and the CO_2_ emissions of blast furnace gas combustion (IPCC) have the lowest value, only accounting for about 23.33% of that from the MNRE and UN-Russia (shown in Fig. 7).

### Limitations and future work

There are several limitations of our emission dataset. First, the activity data used to calculate energy-related emissions cover only large and medium companies. The missing data makes our inventories incomplete. In the future, we will explore the data for all companies to construct more comprehensive emission inventories of Russia and its constituent entities. Second, the process-related emissions only consider the emission generated from the cement production process. In the future, other process-related emissions will be included, such as iron and steel production, glass production and ammonia production, which can further improve the accuracy of the datasets. Third, due to data unavailability, CO_2_ emissions from 2005 to 2016 only show the emissions by energy types with emissions by sectors missing. In future work, we will further explore the sectoral energy data during this period or downscale to the sectoral level based on economic and demographic indicators.

## Usage Notes

This emission dataset can facilitate the academic studies on Russia’s emission patterns and mitigation strategies. The detailed emission inventories can be used to analyse CO_2_ emissions by sectors and energy types, such as the driving factors of CO_2_ emissions, emission reduction potential, emission efficiency, shadow price of CO_2_ emissions, emission reduction cost, and emission prediction. Apart from the energy-related emission analysis, process-related emissions can be used to investigate the emission characteristics and reduction strategies of cement industry.

These emission inventories are a long time-series dataset and cover both Russia and its 82 constituent entities, which can be used to study the emission characteristics over time and space. Therefore, emission-related study at the global, national, and subnational levels can be carried out and some comparisons can be made to gain more insights.

## Supplementary information

Supplementary File 1

## Data Availability

The Python Code used to draw Fig. 3 and Fig. 4 is published at Supplementary File 1 to show how the data can be loaded and visualized.

## Online-only Tables

**Online-only Table 1:** Economic sectors. The first column indicates the number of sectors contained in this study after excluding the double counting sectors and there are 89 sectors in total.

No.	No.	Socio-economic sectors	Categories
	**A**	**Agriculture, forestry and fishing**	Primary industry
	01	Crop and animal production, hunting and related service activities	
1		Crop production, hunting and related services	
	01.4	Animal production	
2	01.41	Raising of dairy cattle	
3	01.42	Raising of other cattle and buffaloes	
4	01.43	Raising of horses and other equines	
5	01.45	Raising of sheep and goats	
6	01.46	Raising of swine/pigs	
7	01.47	Raising of poultry	
8	01.49	Raising of other animals	
9	01.5	Mixed farming	
10	02	Forestry and logging	
11	03	Fisheries and aquaculture	
	**B**	**Mining and quarrying**	Mining and quarrying
	05	Mining of coal and lignite	
12	05.1	Mining of hard coal	
13	05.2	Mining of lignite	
	06	Extraction of crude petroleum and natural gas	
14	06.1	Extraction of crude petroleum	
15	06.2	Extraction of natural gas	
16	07	Mining of metal ore	
	08	Other mining and quarrying	
17	08.1	Quarrying of stone, sand and clay	
18	08.9	Mining and quarrying n.e.c	
	09	Mining support service activities	
19	09.1	Support activities for petroleum and natural gas extraction	
20	09.9	Support activities for other mining and quarrying	
	**C**	**Manufacturing**	
21	10	Manufacture of food products	Food products
22	11	Manufacture of beverages	
23	12	Manufacture of tobacco products	
24	13	Manufacture of textiles	Textile and leather products
25	14	Manufacture of wearing apparel	
26	15	Manufacture of leather and related products	
27	16	Manufacture of wood and of products of wood and cork, except furniture; manufacture of articles of straw and plaiting materials	Wood, pulp and paper products
28	17	Manufacture of paper and paper products	
29	18	Printing and reproduction of recorded media	
30	31	Manufacture of furniture	
	19	Manufacture of coke and refined petroleum products	Coke and refined petroleum products
31	19.1	Manufacture of coke oven products	
32	19.2	Manufacture of refined petroleum products	
33	19.3	Manufacture of coal, anthracite and brown coal (lignite) and thermal coals	
34	20	Manufacture of chemicals and chemical products	Chemical, rubber and plastic products
35	21	Manufacture of basic pharmaceutical products and pharmaceutical preparations	
36	22	Manufacture of rubber and plastic products	
37	23	Manufacture of other non-metallic mineral products	Other non-metallic mineral products
38	24	Manufacture of basic metals	Basic and fabricated metal products
39	25	Manufacture of fabricated metal products, except machinery and equipment	
40	26	Manufacture of computer, electronic and optical products	Electrical and optical products
41	27	Manufacture of electrical equipment	
42	28	Manufacture of machinery and equipment n.e.c	Transport and other machinery equipment
43	29	Manufacture of motor vehicles, trailers and semi-trailers	
44	30	Manufacture of other transport equipment	
45	32	Other manufacturing	
46	33	Repair and installation of machinery and equipment	
	**D**	**Electricity, gas, steam and air conditioning supply**	Electricity, gas, steam, air conditioning, water and waste
	35	Electricity, gas, steam and air conditioning supply	
47	35.1	Electric power generation, transmission and distribution	
48	35.11	Production of electricity	
49		Transmission, distribution and trade of electricity	
	35.2	Manufacture of gas; distribution of gaseous fuels through mains	
50	35.21	Manufacture of gas	
51		Gas distribution and trade	
	35.3	Steam and air conditioning supply	
	35.30	Steam and air conditioning supply	
52	35.30.1	Steam and air conditioning supply	
53		Transmission, distribution and trade of steam and hot water; Maintenance of thermal network and boiler room	
	**E**	**Water supply; sewerage, waste management and remediation activities**	
54	36	Water collection, treatment and supply	
55	37	Sewerage	
56	38	Waste collection, treatment and disposal activities; materials recovery	
57	39	Remediation activities and other waste management services	
	**F**	**Construction**	Construction
58	41	Construction of buildings	
	42	Civil engineering	
59	42.1	Construction of roads and railways	
60		Construction of other civil projects	
	43	Specialized construction activities	
	43.1	Demolition and site preparation	
61		Demolition and site preparation	
62	43.13	Test drilling and boring	
63		Other construction works	
	**G**	**Wholesale and retail trade; repair of motor vehicles and motorcycles**	Wholesale, retail and repair services
64	45	Wholesale and retail trade and repair of motor vehicles and motorcycles	
	46	Wholesale trade, except for motor vehicles and motorcycles	
65		Non-specialized wholesale trade	
	46.7	Other specialized wholesale	
	46.71	Wholesale of solid, liquid and gaseous fuels and related products	
66	46.71.1	Wholesale of solid fuels	
67	46.71.2	Wholesale of motor fuel, including aviation gasoline	
68	46.71.3	Wholesale of oil	
69	46.71.4	Wholesale of natural gas	
70	46.71.5	Wholesale of liquefied petroleum gases	
71	46.71.6	Wholesale of other fuel types and related products	
72		Wholesale trade of other specialized products	
73	47	Retail trade, except of motor vehicles and motorcycles	
	**H**	**Transportation and storage**	Transport and communication services
	49	Land transport and transport via pipelines	
74	49.1	Passenger rail transport, interurban	
75	49.2	Freight rail transport	
76	49.3	Other passenger land transport	
77	49.4	Freight transport by road and removal services	
	49.5	Transport via pipeline	
78	49.50.1	Pipeline transportation of oil and petroleum products	
79	49.50.2	Pipeline transport of gas	
80	49.50.3	Pipeline transport of other products	
81	50	Water transport	
82	51	Air transport	
83	52	Warehousing and support activities for transportation	
84	53	Postal and courier activities	
85	**J**	**Information and communication**	
86	**P**	**Education**	Other services
	**Q**	**Human health and social work activities**	
87	86	Health service activities	
88		Social service activities	
89	**S**	**Other service activities**	

*Note:* The second column indicates the code of sectors according to the Federal Agency for Technical Regulation and Metrology (Rosstandart).

**Online-only Table 2:** 82 constituent entities of Russia. We use the abbreviation name of the 82 constituent entities based on the standard of International Organization for Standardization (ISO 3166-2).

No.	Constituent entity	Abbr.	No.	Constituent entity	Abbr.
1	Belgorod Region	BEL	43	Stavropol Territory	STA
2	Bryansk Region	BRY	44	Republic of Bashkortostan	BA
3	Vladimir Region	VLA	45	Republic of Mari El	ME
4	Voronezh Region	VOR	46	Republic of Mordovia	MO
5	Ivanovo Region	IVE	47	Republic of Tatarstan	TA
6	Kaluga Region	KLU	48	Udmurtian Republic	UD
7	Kostroma Region	KOS	49	Chuvash Republic	CU
8	Kursk Region	KRS	50	Perm Territory	PER
9	Lipetsk Region	LIP	51	Kirov Region	KIR
10	Moscow Region	MOS	52	Nizhny Novgorod Region	NIZ
11	Orel Region	ORL	53	Orenburg Region	ORE
12	Ryazan Region	RYA	54	Penza Region	PNZ
13	Smolensk Region	SMO	55	Samara Region	SAM
14	Tambov Region	TAM	56	Saratov Region	SAR
15	Tver Region	TVE	57	Ulyanovsk Region	ULY
16	Tula Region	TUL	58	Kurgan Region	KGN
17	Yaroslavl Region	YAR	59	Sverdlovsk Region	SVE
18	Moscow city	MOW	60	Tyumen Region	TYU
19	Republic of Karelia	KR	60.1	Khanty–Mansi Autonomous Okrug	KHM
20	Komi Republic	KO	60.2	Yamalo-Nenets Autonomous Okrug	YAN
21	Arkhangelsk Region	ARK	60.3	Tyumen Region less autonomous areas	-
21.1	Nenets Autonomous Okrug	NEN	61	Chelyabinsk Region	CHE
21.2	Arkhangelsk Region less autonomous area	-	62	Republic of Buryatia	BU
22	Vologda Region	VLG	63	Republic of Altay	AL
23	Kaliningrad Region	KGD	64	Republic of Tuva	TY
24	Leningrad Region	LEN	65	Republic of Khakassia	KK
25	Murmansk Region	MUR	66	Altay Territory	ALT
26	Novgorod Region	NGR	67	Trans-Baikal Territory	ZAB
27	Pskov Region	PSK	68	Krasnoyarsk Territory	KYA
28	St. Petersburg city	SPE	69	Irkutsk Region	IRK
29	Republic of Adygeya	AD	70	Kemerovo Region	KEM
30	Republic of Kalmykia	KL	71	Novosibirsk Region	NVE
31	Republic of Crimea	CRI	72	Omsk Region	OMS
32	Krasnodar Territory	KDA	73	Tomsk Region	TOM
33	Astrakhan Region	AST	74	Republic of Sakha (Yakutia)	SA
34	Volgograd Region	VGG	75	Kamchatka Territory	KAM
35	Rostov Region	ROS	76	Primorye Territory	PRI
36	Sevastopol city	SEV	77	Khabarovsk Territory	KHA
37	Republic of Daghestan	DA	78	Amur Region	AMU
38	Republic of Ingushetia	IN	79	Magadan Region	MAG
39	Kabardino-Balkarian Republic	KB	80	Sakhalin Region	SAK
40	Karachayevo-Chircassian Republic	KC	81	Jewish Autonomous Region	YEV
41	Republic of North Ossetia – Alania	SE	82	Chukotka Autonomous Area	CHU
42	Chechen Republic	CE			

*Note: *Because of Ukraine’s political crisis, the abbreviation names of the Republic of Crimea and Sevastopol city are not available, we use CRI and SEV to denote them, respectively.

**Online-only Table 3:** CO_2_ emissions of 82 constituent entities during 2005–2019, in million tonnes.

	2005	2007	2009	2011	2013	2015	2017	2019		2005	2007	2009	2011	2013	2015	2017	2019
BEL	3.9	5.1	7.4	8.9	9.3	9.7	10.1	12.7	CE	—	—	0.3	0.4	0.6	0.4	0.5	1.1
BRY	4.4	4.3	4.0	4.7	4.3	4.0	3.7	3.8	STA	15.4	15.1	14.8	16.3	14.5	16.2	14.0	12.0
VLA	5.8	5.2	5.2	5.4	4.2	5.3	4.9	4.4	BA	38.4	37.3	33.1	38.1	37.6	36.1	47.5	51.4
VOR	7.2	7.2	6.8	7.2	6.7	3.8	8.9	8.5	ME	2.8	2.7	2.4	2.6	2.8	2.5	2.2	2.3
IVE	4.5	4.5	4.4	4.0	3.8	3.3	3.4	3.1	MO	3.8	3.7	3.5	4.5	4.5	4.1	3.8	3.9
KLU	3.0	3.0	3.1	3.1	3.5	4.5	4.0	3.9	TA	29.9	29.0	28.9	34.7	31.9	31.8	33.8	38.5
KOS	8.6	8.8	8.2	9.1	9.1	9.0	9.5	9.7	UD	10.2	10.3	9.6	9.5	10.4	9.6	9.2	7.9
KRS	5.3	5.4	4.2	4.5	4.3	4.5	4.1	4.2	CU	4.2	4.5	3.9	4.5	4.3	3.9	3.7	3.4
LIP	63.7	70.2	65.3	71.9	80.9	89.4	91.3	83.2	PER	42.6	41.8	34.7	39.2	35.6	34.7	34.1	33.2
MOS	43.6	43.1	39.1	42.6	40.6	42.6	45.7	32.2	KIR	7.4	7.4	7.2	7.3	5.6	7.5	5.8	5.4
ORL	2.8	2.6	2.0	2.5	2.8	2.7	2.9	2.7	NIZ	20.0	20.9	20.1	20.8	19.3	20.5	18.2	16.6
RYA	8.8	8.4	7.9	8.5	12.1	5.8	5.1	4.9	ORE	29.9	30.6	30.7	29.4	29.0	39.6	26.1	29.3
SMO	6.2	6.1	5.7	6.0	5.7	5.9	4.5	4.5	PNZ	16.4	15.5	4.0	17.4	14.6	15.7	18.0	19.8
TAM	4.4	3.4	3.1	3.2	3.1	3.3	3.3	3.3	SAM	27.0	27.8	25.5	36.8	29.7	31.6	32.1	28.5
TVE	9.4	11.5	9.8	11.7	11.4	9.5	8.8	8.1	SAR	12.7	12.4	11.3	11.9	10.9	10.3	10.2	10.9
TUL	27.4	28.5	23.3	26.0	26.8	25.7	27.4	28.0	ULY	8.4	8.5	7.6	8.3	6.6	6.7	6.6	5.9
YAR	11.1	10.9	10.6	12.0	11.9	12.5	12.4	12.2	KGN	3.2	3.3	3.8	4.2	4.9	4.3	4.0	4.0
MOW	65.8	68.2	53.6	67.6	63.0	64.5	73.6	79.0	SVE	102.1	107.5	95.6	105.5	106.4	105.2	89.9	86.6
KR	4.6	4.0	3.8	3.9	3.8	3.6	3.8	4.2	TYU	113.4	118.5	115.8	137.4	139.6	132.6	127.9	137.7
KO	18.4	18.6	19.2	19.3	20.7	21.8	21.7	21.5	CHE	122.9	125.3	113.6	128.5	126.3	125.7	120.8	119.3
ARK	21.5	19.9	19.8	18.2	15.5	17.3	20.2	21.8	BU	13.1	13.2	15.9	15.2	10.5	12.4	10.7	12.0
VLG	48.6	56.7	49.7	57.2	62.1	53.5	60.8	54.8	AL	0.3	0.3	0.5	0.9	1.0	0.7	0.3	0.3
KGD	2.8	3.5	3.8	5.1	5.0	5.6	5.5	5.3	TY	0.8	0.7	0.8	0.8	0.8	0.8	2.4	1.2
LEN	14.4	11.5	15.5	17.4	17.8	19.1	23.9	28.1	KK	5.0	4.5	5.2	4.6	3.7	5.5	4.7	4.9
MUR	8.7	8.4	7.8	9.2	8.4	7.0	7.4	7.8	ALT	11.8	12.2	11.7	12.0	15.9	11.6	12.2	9.7
NGR	4.2	4.1	3.9	4.3	6.3	5.3	5.6	5.0	ZAB	12.8	12.4	15.5	14.0	14.4	13.8	11.6	12.6
PSK	2.7	2.6	3.0	3.0	2.6	2.1	2.6	2.3	KYA	62.2	60.0	60.6	59.4	59.4	60.6	55.3	48.6
SPE	20.0	21.3	29.7	32.2	32.6	34.8	38.7	39.0	IRK	38.5	38.6	36.1	35.4	33.7	33.3	28.2	27.9
AD	1.1	0.8	0.6	0.5	0.1	0.3	1.0	0.9	KEM	56.0	57.7	51.2	52.5	61.7	52.2	58.8	58.6
KL	0.4	0.3	0.3	0.3	0.2	0.2	0.3	0.4	NVE	20.6	17.4	19.8	20.8	24.8	20.8	22.1	20.8
CRI	—	—	—	—	—	2.0	4.1	5.2	OMS	21.7	21.3	19.1	20.0	22.5	21.8	19.6	19.2
KDA	14.3	15.8	14.7	16.5	17.9	19.3	21.1	21.3	TOM	9.4	9.1	9.4	9.5	9.9	8.9	7.5	8.4
AST	7.3	6.7	7.4	7.7	7.0	7.5	7.3	7.8	SA	16.5	45.0	15.4	16.2	14.0	17.1	15.6	17.7
VGG	16.3	16.0	13.2	16.9	18.0	18.9	15.6	14.7	KAM	3.0	3.0	3.0	2.9	3.0	2.8	3.3	3.3
ROS	21.1	21.4	20.1	22.2	21.3	18.9	21.2	18.3	PRI	28.9	24.9	25.2	23.2	20.6	21.0	19.6	20.3
SEV	—	—	—	—	—	0.3	1.3	0.5	KHA	14.5	12.7	10.8	11.1	13.8	13.0	14.1	13.8
DA	0.7	0.5	0.8	0.5	0.6	0.7	0.7	1.8	AMU	12.0	10.4	9.7	9.1	8.8	10.1	9.9	10.4
IN	0.1	0.5	0.1	0.1	0.1	0.1	0.1	0.1	MAG	1.6	1.4	1.4	1.6	2.7	1.6	1.8	2.1
KB	1.1	1.0	1.0	0.8	0.8	0.9	0.9	0.8	SAK	8.9	9.1	8.5	7.7	4.7	5.0	6.1	6.9
KC	1.4	1.9	1.4	1.6	1.6	1.6	1.4	1.5	YEV	1.3	1.3	1.0	0.8	0.9	0.8	0.6	0.6
SE	1.1	1.2	1.1	0.7	0.9	1.0	0.8	0.8	CHU	2.4	3.5	2.0	2.5	1.0	1.3	1.0	0.8

**Online-only Table 4:** Comparisons of emission factors from different sources.

NCV (TJ/1000 tonnes (mln m3))					
No.	Energy types	MNRE-this study	IPCC	SRO	UN-Russia
1	Gasoline	43.70	44.43	44.21	43.67
2	Fuel oil	40.20	40.40	41.15	40.15
3	Diesel	42.50	43.00	42.80	42.50
4	Bunker fuel	41.12	41.44	41.15	41.91
5	Marine fuel	41.90	40.57	40.28	41.03
6	Liquefied propane and butane	46.00	46.40	47.31	46.01
7	Other petroleum products	41.90	40.00	40.19	41.91
8	Artificial coke gas	16.70	18.80	16.73	16.71
9	Blast furnace gas	12.60	3.00	5.75	12.60
10	Combustible natural gas	33.80	34.40	34.78	33.82
11	Associated petroleum gas	33.80	47.30	—	—
12	Fuelwood	6.16	10.90	10.22	7.80
13	Household boiler fuel	42.50	40.19	42.54	42.50
14	Peat	10.00	9.76	10.00	9.96
15	Peat briquettes and semi-briquettes	17.60	9.76	10.00	9.96
16	Other solid fuel	13.61	—	—	—
17	Coal	—^3^	28.20	17.62	20.05
Carbon Content (t C/TJ)					
1	Gasoline	18.90	18.90	19.13	18.90
2	Fuel oil	21.10	21.10	20.84	21.11
3	Diesel	20.20	20.20	19.98	20.21
4	Bunker fuel	20.74	20.74	20.84	21.11
5	Marine fuel	21.10	21.88	21.98	22.27
6	Liquefied propane and butane	17.30	16.80	17.20	17.21
7	Other petroleum products	20.00	20.00	20.00	19.99
8	Artificial coke gas	12.10	12.10	13.00	12.11
9	Blast furnace gas	71.00	70.91	66.00	70.91
10	Combustible natural gas	14.80	15.30	15.04	14.84
11	Associated petroleum gas	16.50	17.20	-	-
12	Fuelwood	30.56	30.50	29.48	30.55
13	Household boiler fuel	21.10	21.11	20.29	21.11
14	Peat	28.90	28.91	29.00	28.91
15	Peat briquettes and semi-briquettes	28.90	28.91	29.00	28.91
16	Other solid fuel	27.85	27.27	—	—
17	Coal	—^3^	25.80	25.58	25.69
Oxygenation efficiency					
1	Gasoline	0.99	0.99	0.99	1.00
2	Fuel oil	0.99	0.99	0.99	0.99
3	Diesel	0.99	0.99	0.99	0.99
4	Bunker fuel	0.99	0.99	0.99	0.99
5	Marine fuel	0.99	0.99	0.99	0.99
6	Liquefied propane and butane	0.99	0.99	0.99	0.99
7	Other petroleum products	0.99	0.99	0.99	0.99
8	Artificial coke gas	1.00	0.99	1.00	1.00
9	Blast furnace gas	1.00	0.98	1.00	1.00
10	Combustible natural gas	1.00	1.00	1.00	1.00
11	Associated petroleum gas	0.99	0.99	0.99	1.00
12	Fuelwood	0.98	1.00	0.98	0.98
13	Household boiler fuel	0.99	0.99	0.98	0.98
14	Peat	0.98	0.99	0.99	0.98
15	Peat briquettes and semi-briquettes	0.98	0.99	0.99	0.98
16	Other solid fuel	1.00	1.00	—	—
17	Coal	—^3^	0.98	0.98	0.98
t CO_2_/(tonne,1000 m3)					
1	Gasoline	3.00	3.05	3.07	3.02
2	Fuel oil	3.08	3.09	3.11	3.09
3	Diesel	3.12	3.15	3.10	3.13
4	Bunker fuel	3.10	3.12	3.11	3.21
5	Marine fuel	3.21	3.22	3.21	3.32
6	Liquefied propane and butane	2.89	2.86	2.95	2.87
7	Other petroleum products	3.04	2.90	2.92	3.04
8	Artificial coke gas	0.74	0.83	0.79	0.74
9	Blast furnace gas	3.28	0.76	1.38	3.28
10	Combustible natural gas	1.83	1.91	1.91	1.84
11	Associated petroleum gas	2.02	2.95	—	—
12	Fuelwood	0.68	1.22	1.08	0.86
13	Household boiler fuel	3.26	3.11	3.10	3.22
14	Peat	1.04	1.03	1.05	1.03
15	Peat briquettes and semi-briquettes	1.83	1.03	1.05	1.03
16	Other solid fuel	1.39	1.16	—	—
17	Coal	–^3^	2.61	1.62	1.86

^3^Please refer to Table 1 which includes 29 different types of coal.
