# Supplementary material for: CO2 emission accounts of Russia’s constituent entities 2005–2019
Source: Sci Data. 2021 Jul 13;8:172. doi: 10.1038/s41597-021-00966-z (PMC8277808; doi:10.1038/s41597-021-00966-z)
Supplement: Supplementary file 1 [file 41597_2021_966_MOESM1_ESM.docx]

### Code availability

The Python Code used to draw Fig. 3 and 4 is published below to show how the data can be loaded and visualized.

*#####Code for drawing Fig. 3
#####Step 1: Import library
# -*- coding: utf-8 -*-*import matplotlib.pyplot as plt
*#####Step 2: Import data. The data are included inside the code to ensure working without external files.
Year = ['2005','2006','2007','2008','2009','2010','2011','2012','2013','2014','2015','2016','2017','2018','2019']
fuel1=[515.7,524.3,533.7,555.0,505.0,564.8,580.7,573.3,543.2,571.5,554.1,564.3,573.0,589.4,575.1]
fuel2=[49.3,50.5,51.1,51.5,47.4,52.1,53.5,53.2,56.7,50.3,51.5,50.4,51.4,54.2,56.4]
fuel3=[14.7,16.5,16.5,15.7,16.9,19.2,18.9,18.8,17.2,17.9,17.7,18.2,10.6,10.4,10.3]
fuel4=[217.0,240.4,245.6,222.2,207.4,235.6,235.5,243.3,237.4,246.9,251.2,249.5,248.9,247.7,242.5]
fuel5=[336.9,344.1,337.7,345.4,313.8,297.7,286.5,290.2,296.5,258.6,259.1,245.4,258.3,252.4,241.2]
fuel6=[60.5,65.6,72.6,70.3,61.7,69.1,69.1,72.4,66.3,65.6,65.3,64.1,63.7,62.5,60.6]
fuel7=[2.2,2.2,1.7,1.3,1.3,1.7,1.5,1.4,0.9,1.2,0.7,0.9,0.4,0.4,0.3]
fuel8=[86.9,89.9,94.6,98.4,109.3,119.6,123.8,126.3,140.2,144.5,147.0,148.5,157.3,141.4,155.2]
fuel9=[60.9,64.8,63.9,67.1,60.0,62.4,70.2,70.9,67.5,67.0,68.5,66.1,73.3,73.9,77.7]
fuel10=[58.1,58.6,44.5,41.6,39.8,39.1,34.7,30.0,25.4,23.3,22.2,27.2,19.6,18.7,18.5]
fuel11=[46.8,47.5,47.2,49.8,45.9,49.3,52.4,51.8,44.4,55.7,58.0,56.2,61.0,66.3,67.7]
fuel12=[1.0,1.0,1.0,1.1,1.0,1.0,1.1,1.1,1.1,1.5,1.7,1.8,2.8,3.8,3.6]
fuel13=[3.5,3.4,4.3,4.3,4.4,4.3,3.9,3.5,4.4,3.5,2.9,2.9,3.6,2.1,2.1]
fuel14=[2.1,2.2,2.1,2.2,2.3,2.4,3.1,2.8,2.7,2.4,2.3,2.2,2.0,3.5,2.5]
fuel15=[0.1,0.1,0.1,0.0,0.0,0.0,0.0,0.0,0.1,0.0,0.0,0.0,0.1,0.1,0.1]
fuel16=[5.4,5.7,2.2,2.4,3.4,4.0,4.3,4.4,3.9,4.4,4.8,5.2,10.1,10.7,12.3]
fuel17=[1.4,1.3,1.1,1.2,0.9,0.7,0.7,0.6,0.6,0.5,0.5,0.6,0.6,0.4,0.4]
fuel18=[23.9,26.3,29.7,25.3,21.2,24.3,26.9,28.2,29.2,28.3,24.7,21.8,21.9,21.5,23.0]
#####Step 3:Draw the stacked area chart. The parameters can be set based on preference, such as color and fontsize*plt.figure(figsize=(9,6))
plt.stackplot(Year,fuel1,fuel2,fuel3,fuel4,fuel5,fuel6,fuel7,fuel8,fuel9,fuel10,fuel11,fuel12,fuel13,fuel14,fuel15,fuel16,fuel17,fuel18,colors=['navy','royalblue','Olivedrab','yellowgreen','darkgoldenrod','tan','olive','darkorange','orange','gold','khaki','palegoldenrod','beige','ivory','hotpink','palevioletred','pink','darkgrey'])
plt.xlabel('Year',fontsize=18,family='Times New Roman')
plt.ylabel('CO_2_ emissions',fontsize=18,family='Times New Roman')
plt.xticks(fontsize=18,family='Times New Roman')
plt.yticks(fontsize=18,family='Times New Roman')
plt.legend(['Combustible natural gas','Associated petroleum gas','Artificial coke gas','Blast furnace gas','Coal','Peat','Peat briquettes and semi-briquettes','Gasoline','Diesel','Fuel oil','Other petroleum products','Bunker fuel','Liquefied propane and butane','Marine fuel','Fuelwood','Other solid fuel','Household boiler fuel','Process'],fontsize = 18)
plt.show()

*#####Code for drawing Fig. 4
#####Step 1: Import libraries
# -*- coding: utf-8 -*-*import matplotlib.pyplot as plt
import pandas as pd
*#####Step 2: The data are included inside the code to ensure working without external files; Alternative simple way is running this code (data = pd.read_csv(filepath)) to import data.*raw_data='''Entity;Primary industry;Mining and quarrying;Food products;Textile and leather products;Wood,pulp and paper products;Coke and refined petroleum products;Chemical, rubber and plastic products;Other non-metallic mineraal products;Basic and fabricated metal products;Electrical and optical products;Transport and other machinary equipment;Electricity, gas, steam,air cond.;Construction;Wholesale, retail and repaire services;Transport and communication services;Other services\n
TYU;0.5 ;32.9 ;0.1 ;0.0 ;0.0 ;7.5 ;0.6 ;0.0 ;0.1 ;0.0 ;2.0 ;48.2 ;2.1 ;5.0 ;37.8 ;0.5 \nCHE;0.6 ;0.9 ;0.3 ;0.0 ;0.0 ;0.5 ;0.6 ;92.2 ;3.2 ;0.0 ;0.7 ;18.2 ;0.4 ;0.2 ;1.0 ;1.2 \n
SVE;1.0 ;1.3 ;0.4 ;0.0 ;0.1 ;0.0 ;0.4 ;36.4 ;4.1 ;0.2 ;2.7 ;34.6 ;0.9 ;0.8 ;2.6 ;2.1 \nLIP;0.9 ;0.0 ;0.7 ;0.0 ;0.0 ;0.0 ;10.3 ;68.2 ;0.7 ;0.0 ;0.1 ;1.7 ;0.0 ;0.1 ;0.7 ;0.2 \n
MOS;0.1 ;0.1 ;0.9 ;0.0 ;0.9 ;1.3 ;0.4 ;0.2 ;0.1 ;0.3 ;1.1 ;37.4 ;3.5 ;11.0 ;16.0 ;4.6 \nKEM;0.4 ;5.4 ;0.4 ;0.0 ;0.0 ;2.4 ;0.9 ;14.3 ;1.9 ;0.0 ;0.8 ;27.4 ;0.5 ;2.1 ;2.5 ;0.6 \n
VLG;0.2 ;0.0 ;0.1 ;0.0 ;0.3 ;0.0 ;0.0 ;46.8 ;0.1 ;0.0 ;0.0 ;3.5 ;0.2 ;0.0 ;3.3 ;0.1 \nBA;0.6 ;0.4 ;0.5 ;0.0 ;0.1 ;17.4 ;4.4 ;0.1 ;2.3 ;0.0 ;0.2 ;20.7 ;0.8 ;2.3 ;1.9 ;0.4 \n
KYA;1.2 ;0.4 ;0.1 ;0.0 ;0.4 ;0.0 ;0.0 ;10.2 ;1.1 ;0.0 ;0.3 ;28.1 ;0.6 ;0.6 ;5.2 ;0.8 \nSPE;0.1 ;0.0 ;3.1 ;0.0 ;0.0 ;0.0 ;0.3 ;8.5 ;0.2 ;0.1 ;1.8 ;18.0 ;0.9 ;1.5 ;4.1 ;0.4\n
'''

raw_data2=raw_data.split('\n')
raw_data3=[]
for line in raw_data2:
 if len(line)>0:
 raw_data3.append(line.split(';'))
data=pd.DataFrame(raw_data3[1:],columns=raw_data3[0])
for col in data.columns[1:]:
 data[col] = data[col].astype('float64')
for i,col_name in enumerate(data.columns[0:]):
 locals()['sector_'+str(i)]=col_name
fig, ax = plt.subplots(figsize=(10,6))
*#####Stpe 3: Draw the stacked bar chart. The parameters can be set based on preference, such as color, bar width, and fontsize.*width=0.35
ax.bar(data['Entity'], data[sector_1], width, label=sector_1,color='navy')
ax.bar(data['Entity'], data[sector_2], width, bottom=data[sector_1],label=sector_2,color='royalblue')
ax.bar(data['Entity'], data[sector_3], width, bottom=data[sector_1]+data[sector_2],label=sector_3,color='cornflowerblue')
ax.bar(data['Entity'], data[sector_4], width, bottom=data[sector_1]+data[sector_2]+data[sector_3],label=sector_4,color='lightsteelblue')
ax.bar(data['Entity'], data[sector_5], width, bottom=data[sector_1]+data[sector_2]+data[sector_3]+data[sector_4],label=sector_5,color='lavender')
ax.bar(data['Entity'], data[sector_6], width, bottom=data[sector_1]+data[sector_2]+data[sector_3]+data[sector_4]+data[sector_5],label=sector_6,color='darkorange')
ax.bar(data['Entity'], data[sector_7], width, bottom=data[sector_1]+data[sector_2]+data[sector_3]+data[sector_4]+data[sector_5]+data[sector_6],label=sector_7,color='orange')
ax.bar(data['Entity'], data[sector_8], width, bottom=data[sector_1]+data[sector_2]+data[sector_3]+data[sector_4]+data[sector_5]+data[sector_6]+data[sector_7],label=sector_8,color='navajowhite')
ax.bar(data['Entity'], data[sector_9], width, bottom=data[sector_1]+data[sector_2]+data[sector_3]+data[sector_4]+data[sector_5]+data[sector_6]+data[sector_7]+data[sector_8],label=sector_9,color='gold')
ax.bar(data['Entity'], data[sector_10], width, bottom=data[sector_1]+data[sector_2]+data[sector_3]+data[sector_4]+data[sector_5]+data[sector_6]+data[sector_7]+data[sector_8]+data[sector_9],label=sector_10,color='khaki')
ax.bar(data['Entity'], data[sector_11], width, bottom=data[sector_1]+data[sector_2]+data[sector_3]+data[sector_4]+data[sector_5]+data[sector_6]+data[sector_7]+data[sector_8]+data[sector_9]+data[sector_10],label=sector_11,color='lightyellow')
ax.bar(data['Entity'], data[sector_12], width, bottom=data[sector_1]+data[sector_2]+data[sector_3]+data[sector_4]+data[sector_5]+data[sector_6]+data[sector_7]+data[sector_8]+data[sector_9]+data[sector_10]+data[sector_11],label=sector_12,color='yellowgreen')
ax.bar(data['Entity'], data[sector_13], width, bottom=data[sector_1]+data[sector_2]+data[sector_3]+data[sector_4]+data[sector_5]+data[sector_6]+data[sector_7]+data[sector_8]+data[sector_9]+data[sector_10]+data[sector_11]+data[sector_12],label=sector_13,color='olivedrab')
ax.bar(data['Entity'], data[sector_14], width, bottom=data[sector_1]+data[sector_2]+data[sector_3]+data[sector_4]+data[sector_5]+data[sector_6]+data[sector_7]+data[sector_8]+data[sector_9]+data[sector_10]+data[sector_11]+data[sector_12]+data[sector_13],label=sector_14,color='mediumaquamarine')
ax.bar(data['Entity'], data[sector_15], width, bottom=data[sector_1]+data[sector_2]+data[sector_3]+data[sector_4]+data[sector_5]+data[sector_6]+data[sector_7]+data[sector_8]+data[sector_9]+data[sector_10]+data[sector_11]+data[sector_12]+data[sector_13]+data[sector_14],label=sector_15,color='lavenderblush')
ax.bar(data['Entity'], data[sector_16], width, bottom=data[sector_1]+data[sector_2]+data[sector_3]+data[sector_4]+data[sector_5]+data[sector_6]+data[sector_7]+data[sector_8]+data[sector_9]+data[sector_10]+data[sector_11]+data[sector_12]+data[sector_13]+data[sector_14]+data[sector_15],label=sector_16,color='silver')
ax.set_ylabel('CO_2_ emissions (Million tonnes)',fontsize=12,family='Times New Roman')
ax.set_title('(A) Top 10',fontsize=14,family='Times New Roman')
plt.xticks(fontsize=12,family='Times New Roman')
plt.yticks(fontsize=12,family='Times New Roman')
plt.legend(loc='upper right',fontsize=10)
plt.ylim(0,160)
plt.show()
